# Secure Comparisons of Single Nucleotide Polymorphisms Using Secure Multiparty Computation: Method Development

**DOI:** 10.2196/44700

**Published:** 2023-07-18

**Authors:** Andrew Woods, Skyler T Kramer, Dong Xu, Wei Jiang

**Affiliations:** 1 Department of Electrical Engineering and Computer Science University of Missouri Columbia, MO United States; 2 Christopher S. Bond Life Sciences Center University of Missouri Columbia, MO United States; 3 Institute for Data Science and Informatics University of Missouri Columbia, MO United States

**Keywords:** secure multiparty computation, single nucleotide polymorphism, Variant Call Format, Jaccard similarity

## Abstract

**Background:**

While genomic variations can provide valuable information for health care and ancestry, the privacy of individual genomic data must be protected. Thus, a secure environment is desirable for a human DNA database such that the total data are queryable but not directly accessible to involved parties (eg, data hosts and hospitals) and that the query results are learned only by the user or authorized party.

**Objective:**

In this study, we provide efficient and secure computations on panels of single nucleotide polymorphisms (SNPs) from genomic sequences as computed under the following set operations: union, intersection, set difference, and symmetric difference.

**Methods:**

Using these operations, we can compute similarity metrics, such as the Jaccard similarity, which could allow querying a DNA database to find the same person and genetic relatives securely. We analyzed various security paradigms and show metrics for the protocols under several security assumptions, such as semihonest, malicious with honest majority, and malicious with a malicious majority.

**Results:**

We show that our methods can be used practically on realistically sized data. Specifically, we can compute the Jaccard similarity of two genomes when considering sets of SNPs, each with 400,000 SNPs, in 2.16 seconds with the assumption of a malicious adversary in an honest majority and 0.36 seconds under a semihonest model.

**Conclusions:**

Our methods may help adopt trusted environments for hosting individual genomic data with end-to-end data security.

## Introduction

### Background

With the dramatic decrease in sequencing costs and increase in consumer sequencing organizations, there is no shortage of genomics data. In fact, about 38 million people worldwide had taken a direct consumer genetics test from organizations like 23andMe, Ancestry, and Family Tree DNA by 2021 [1].

The genome is valuable for identifying health risks, predicting drug response, and revealing susceptibility to environmental factors. Genomic data are the foundation of personalized medicine. However, there are many privacy risks involved with access to genomic data. For example, health insurance companies can gain access to this data through genomic databanks via financial compensation, then deny coverage to a potential customer based on their genetic health risks. While the Genetic Information Nondiscrimination Act protects against such discriminatory acts, detecting and enforcing such a law requires effort that could be mitigated if the company never gets the data in the first place. Traditional cryptographic methods may protect against data leakage, but they often prevent legitimate data queries for research and medical purposes. In today’s ever-growing reliance on such data and collaboration, the need for secure computation on genomic data is rapidly growing. Thus, we propose methods in the realm of secure computation, which allows computation on private or sensitive data. We make use of secure computational methods to do secure similarity measures on genetic data without revealing anything about the data itself.

Genetic data is massive, so there are many different methods of expressing that data depending on the application the data is used for. We focus our attention on variants, which are often reported in the Variant Call Format (VCF) file [2] and can refer to substitutions, insertions, and deletions (indels); copy number variations; and others. These variants correlate with relationships between individuals and can be used to identify such relationships. The correlation carries over to properly chosen subsets of these variations, which we refer to as panels. Using these panels, we can perform operations on smaller sets with a fixed and reduced size to compute the similarity between two individuals more efficiently.

### Related Work

There are many other works that discuss secure similarity comparisons of genetic data, typically in the context of approximating edit distance [3-6]. Aziz et al [3] used shingle set intersection as an alternative method of the similarity metric. However, edit distance is not the only method of comparison and lacks much information about the associated genetic material. For example, edit distance is positionally agnostic. Further, computing the edit distance takes O(mn) time for two strings of length m and n. Some other methods discussed performing set operations on variants [7], which aim to answer a different question than our approach. Our approach targets how similar two individuals are without identifying any genes or variants while they aim to identify the specific genes in the sets to help look for causal genes in diseases.

Some other works used variation sets to compare two genomes [6,8], but these works have some limitations. For example, the methods by Çetin et al [8] allow a data owner to outsource their data and securely query the data so that the server learns nothing about the VCF data. The specific operation asks if a small set of variants exists in some VCF data. The act of executing the query provides too much information about the stored VCF data and cannot be used for queries from outsiders. Zhu et al [6] focused on looking at small edit sets (VCFs) from shorter sequences and did not look at whole genome similarity, which is what our approach aims to look at. The method by Mahdi et al [4] aimed to securely compute the Hamming distance to search for the most similar sequences in a database using prefix tree queries. However, the method used a trusted party to encrypt the data and distributes decryption keys to researchers, but the expectation of the existence of a trusted party is not practical.

### General Approach

In our scheme, we make use of secure multiparty computational techniques to allow secret sharing of filtered variation data. These techniques are built to allow a set of parties to compute on joint data without revealing anything about the input other than what can be derived from the output. Such methods typically use secret sharing schemes or homomorphic encryption schemes, which permit users to perform computations on the encrypted data without first decrypting it.

We consider two entities, each with an individual’s genetic data to compare; both parties then agree on a set of important genetic variants through a public panel and ordering of those variants, and encode their genetic data into a binary vector based on the presence of the selected variants in their VCF files. The owners of the binary vectors then secretly share each element in the vector. Each sharing will consist of pieces, and each piece will be given to one of the computing servers. The servers then compute our protocol on the input shares to produce and send output shares to the user, who then reconstructs the shares to get the result.

In this problem domain, the participating parties (ie, individuals or organizations) can be classified into three categories: (1) database owners, (2) users, and (3) service providers (SPs).

- Database owners: These entities could be any health care organization, ancestry company, or genetic profiling company. They provide services for others to query their DNA data but do not want to expose them.
- Users: The users are parties who wish to make queries in the database. They may be individual patients, medical doctors looking for treatment for their patients by means of a similar patient query, other data owners, or researchers from universities.
- Service providers: The SPs can be any organization that offers computational infrastructures, such as Amazon (Amazon Web Service [AWS]), Microsoft (Azure), and Google (Google Cloud Platform [GCP]).

Note that the roles are not mutually exclusive, and a single party may take on multiple roles without compromising the system’s security. For example, a database owner could play the role of one of the SPs and take part in the computation.

**Figure 1:**
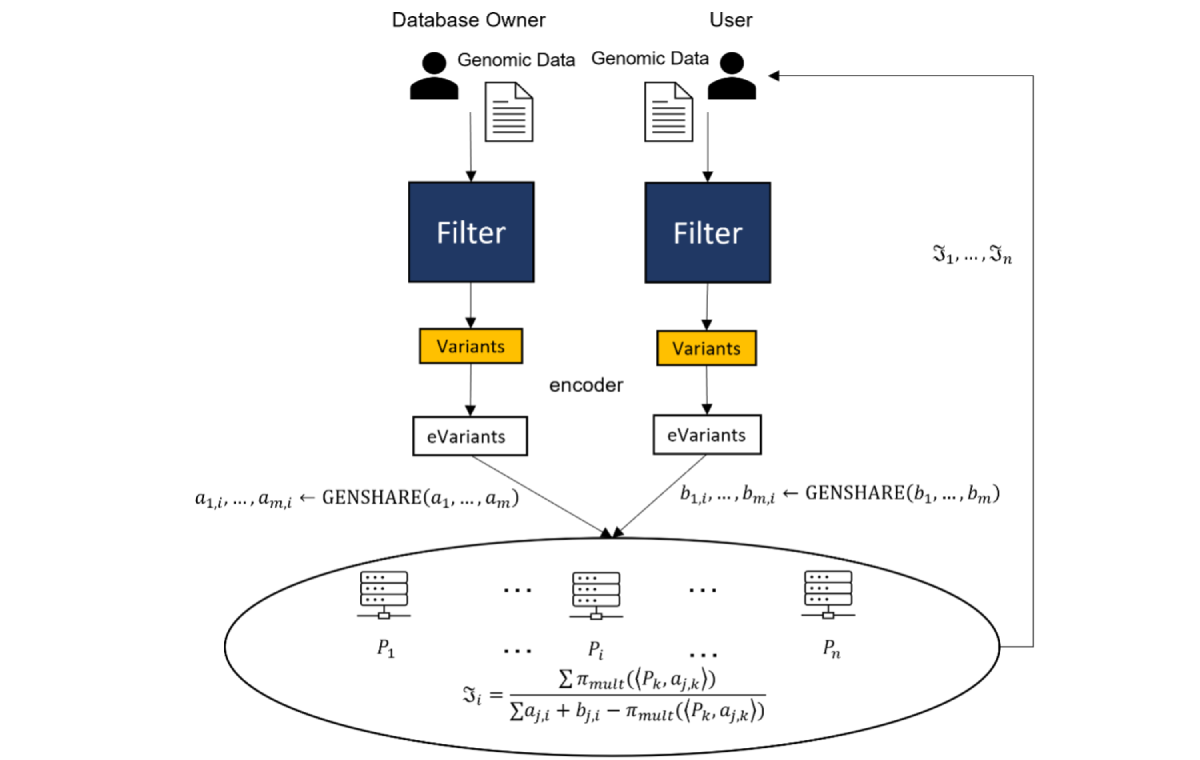
The database owner and the user filter their genomic data for variants and encode them into a binary string (eVariants) of the same length as the selected panel used for comparisons. Elements of the string are 1 if the implicated variant is present or 0 otherwise. They are then secretly shared with the service providers for secure computation. The service providers do not learn anything about the variants because they only receive secret shares of the encoded variants and none of the encoded variants themselves. Next, each player (Pi) computes the secret share (Ji) by using the secret shares received. Each server (Pi) then sends (Ji) to the user. The user then applies these shares to reconstruct the Jaccard similarity (J←RECONSTRUCT(⟨Pi,Ji⟩).

The goal is to provide secure methods to compute set operations on the two input sets of variants, as shown in Figure 1. We include methods to compute the union, intersection, set difference, symmetric difference, and Jaccard similarity on filtered sets of VCFs. We use these methods, for example, to compute the Jaccard similarity between two sets of variants. The protocol is broken up into three phases, the input phase, the computation phase, and the output phase.

- Computation phase: The SPs will perform the specified set operations on the sets of single nucleotide polymorphism (SNPs; or other types of variants) retrieving the sizes of the sets. These sizes can then be used to compute similarity metrics between the two input sequences. These similarity metrics can then be used according to the specific application, such as finding the top *k* matches.
- Output phase: The SPs receive the shares of the output. After this, the SPs can send their final shares to the user so that the user can reconstruct the result.

Figure 1 illustrates the general process when the parties are interested in the Jaccard similarity. The final values *J*_1_, *J*_2_, *J*_3_ held by the servers are seemingly random and, thus, do not individually give any information of the actual Jaccard similarity *J*. However, the full set of values can be used to reconstruct *J*. Precisely, we have *J* ← RECONSTRUCT (‹*P*_1_,*J*_1_›,‹*P*_2_,*J*_2_›,‹*P*_3_,*J*_3_›). The user can learn *J* by having each server *P_i_* send their share *J_i_*, then using the RECONSTRUCT function.

### Contribution

We propose a method to securely compute the Jaccard similarity over two individuals’ filtered set of genetic variants. We use the Multi-Party Secure, Privacy-Preserving, and Decentralized Zeus (MP-SPDZ) framework [9] to test the run time and communication costs of our approach. We also tested our approach on a few different popular secure multiparty computation paradigms considering different adversarial models. We then show that our approach provides useful information about the data when the filtered set is chosen properly. We make use of a highly informative but small SNP panel [10] with 4763 SNPs and VCFs from the Genome in a Bottle (GIAB) data set to show that there exists an SNP panel that can be used to identify familial relationships as an example application of our approach. These results are compared with the genomic comparison software BEDTools [11].

## Methods

### Preliminaries

In this section, we give an overview of the technical background that is needed for our protocols. We start by describing secure multiparty computation—the foundation that enables the execution of our protocols. We then introduce secret sharing and the two types used in the protocols behind the primitive operations in our protocols. We will then discuss the MP-SPDZ framework and how it helped us test the resource requirements behind our protocols.

### Threat Model

Secure computation aims to provide guarantees on the privacy of data and correctness of computation. Any situation that potentially compromises these two guarantees is considered a threat. We will assume that the database owner and the user are both semihonest [12]. This means that the database owner and the user will follow the protocol as prescribed. However, we will consider both honest-but-curious and malicious adversarial models for the computing servers. The protocols we run from the MP-SPDZ framework are based on those assumptions. The computational costs of the protocols are correlated with the assumptions of the behavior of the adversary. For example, if the adversary may do something not prescribed in the protocol, we must use extra measures to ensure that behavior does not cause any compromises to the security guarantees such as correctness or privacy.

### Secure Multiparty Computation

Secure multiparty computation allows a set of *n* parties to compute a function over their private inputs without revealing anything about the input other than what can be derived from the output. There are many works providing methods for secure multiparty computation [13-20]. We adapted the methods provided by the MP-SPDZ framework [9] and its protocols. The protocols we used are Semihonest Oblivious Transfer (Semi-OT), MASCOT, Shamir, and Malicious Shamir (Mal-Shamir). Here, OT in Semi-OT and MASCOT both stand for oblivious transfer used to compute multiplication under additive sharing [19,21].

We give a more detailed description of the methods implemented in MP-SPDZ that are used to execute our protocols.

- Semihonest Shamir (Shamir): A semihonest protocol based on the Shamir sharing scheme [22]. This protocol requires at least three computing parties to be used. It is the semihonest equivalent of the honest-majority maliciously secure protocol Mal-Shamir. This protocol is referred to as “Shamir” in the MP-SPDZ framework.
- Mal-Shamir: A maliciously secure execution of the Shamir protocol. This protocol also requires at least three computing parties and can tolerate a minority of players deviating from the protocol and preserve correctness and privacy. This protocol is referred to as “Mal-Shamir” in the MP-SPDZ framework.
- Semi-OT: A semihonest equivalent of the MASCOT protocol, multiplication is based on the OT protocol. This protocol can be executed with as few as two computing parties. This protocol is referred to as “Semi” in the MP-SPDZ framework.
- MASCOT: A maliciously secure malicious majority protocol. This protocol makes use of oblivious transfer to compute multiplications and can also be executed with as few as two computing parties [19]. This protocol is referred to as “MASCOT” in the MP-SPDZ framework.

These protocols are specifications on how to perform addition and multiplication in a secure setting. We make use of these specifications so that our protocol can be executed securely. All these specifications rely on a notion called secret sharing.

Secret sharing forms the foundation of many secure multiparty computation protocols. A secret sharing is an encoding such that a single element (referred to here as a secret) is used to generate an array via a stochastic function (GENSHARE in 1). In a situation where shares should be turned back into the corresponding value, we use a deterministic function known as RECONSTRUCT.

### Data

We tested our method on a small data set called GIAB as well as a simulated data set that we used to collect larger unspecified use panels that have no specified use only for measuring the scale of complexity growth as the panel size increases.

The simulated portion of the data was simulated according to the method described by Yue and Liti [23] with the GRCh38 Genome Reference Consortium Human Reference 38 from the University of California, Santa Cruz (UCSC) [24] as our reference genome. Specifically, simuG was used to generate a simulated SNP panel of 1 million SNPs and 2 simulated human VCF files with 5 million SNPs in each. The top “k” SNPs from the simulated SNP panel were pulled to test the impact of panel size on the protocol. Importantly, the simulated VCF files were simulated such that they only contained SNPs and no other variants.

In the VCF format, a single SNP element is a tuple containing the chromosome, position, reference allele, and alternate allele—labeled as “Chr,” “Pos,” “Ref,” and “Alt” in the VCF file, respectively. The chromosome represents the chromosome on which the SNP is located, the position gives a direct location on the chromosome, the reference allele is the standard base to be compared within this location, and the alternate allele is the nonreference base found in this location for a specific genome. Thus, standard VCFs will only have entries for observed variants, not all possible variants. Other information is also provided in the file, but it is not important for our method.

We also tested our method on the GIAB data set [25]. This data set consists of two families, an Ashkenazim family and a Chinese family. Both families have a mother, a father, and a son. The Ashkenazim data set’s IDs are HG002, HG003, and HG004 for the son, father, and mother, respectively. The IDs of the Chinese family are HG005, HG006, and HG007 for the son, father, and mother, respectively. To use these VCFs in our method, we made use of an SNP panel [10] with 4763 SNPs.

### Secure Set Operations

In this section, we provide an overview of the secure operations that would be executed to compare two VCFs with SNP panels. Using the variant panel, we created an *m* bit string where each position of the bit string contains a “1” if the implicated variant is in the genome (as indicated by the VCF data) or a “0” otherwise. We can then embed the string into the field *F* to perform a computation that allows us to securely perform set operations based on the array. We are interested in the number of elements in a set from set operations. Though our protocols calculate the size of the sets produced by the set operations, they can be easily modified to produce the set itself by skipping the last step.

The protocols we describe in this section use subprotocols implemented by MP-SPDZ, and we denote these subprotocols with *π*. For example, *π_mult_* multiplies two shared values together. These operations require communication between the parties; thus we use this symbol rather than a simple multiplication symbol. However, affine operations such as addition and multiplication by a constant such as 2 do not require communication and are not written with a subprotocol symbol *π*.

We converted the two sets of genomic variants, *A*, *B* into two *m*-dimensional vectors 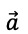 , 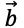. Using these vectors, we can perform elementwise operations on the array to execute set operations and compute any similarity metric that relies on those set operations, such as Jaccard similarity. Here, we provide a formal description of the protocols.

Specifically, let the tuple of the genomic variants be (Chr, Pos, Ref, Alt). To generate the two *m*-dimensional vectors, we select a public panel of variants *S* to filter off and order to. The vector 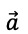 = (*a*_1_, ..., *a_n_*) is defined to be *a_i_* = 1 if the *i*-th SNP (*Chri*, *Posi*, *Ref_i_*, *Alt_i_*) ∈ *S* ∩ *A*; otherwise *a_i_* = 0.



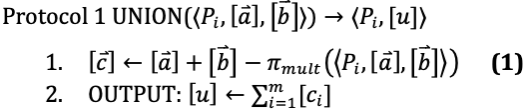



After computing step 1 in UNION, 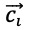 = 1 implies that at least 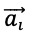 = 1 or 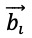 = 1. This means the sum of the elements of the vector 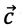 is equal to the size of the union of the two sets *A* and *B*. Thus, in step 2, UNION computes the sharing of the size of the union.



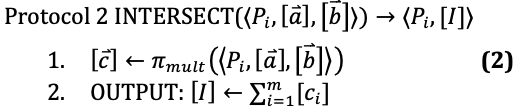



After step 1 in INTERSECT, 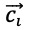 = 1 implies that both 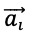 = 1 or 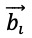 = 1. Thus the sum of the elements in 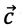 is equal to the size of the intersection of the two sets *A* and *B*. Thus on step 2, the protocol computes a sharing of the size of the intersection of the two sets.



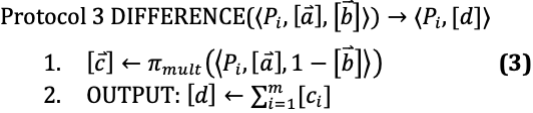



After step 1 in DIFFERENCE, 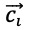 = 1 implies that 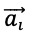 = 1 and 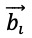 = 0. This corresponds to being the set difference, so computing the sum of 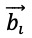 in step 2 gives us a sharing of the size of the set difference *A* \ *B*.



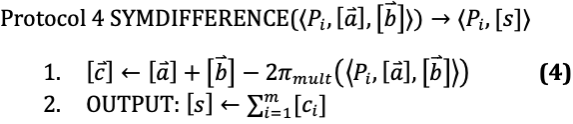



After step 1 in SYMDIFFERENCE, 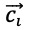 = 1 implies that either 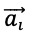 = 1 or 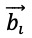 = 1, but not both. Thus, computing the sum in step 2 gives us the size of the symmetric set difference of *A* and *B*.



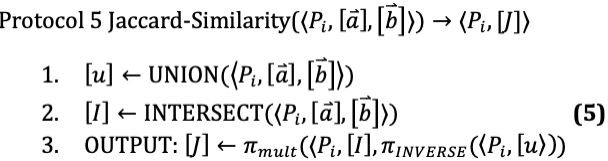



Jaccard similarity is the ratio of the size of the intersection to the union of two sets. Since *A* ∩ *B* ⊆ *A* ∪ *B*, we have |*A* ∩ *B*|≤|*A* ∪ *B*| and that *J* ∈ [0, 1]. Here, we use our previous protocols to first compute the sizes of the union and intersection, and we use the results to compute the Jaccard similarity.

### Network Setup

**Figure 2:**
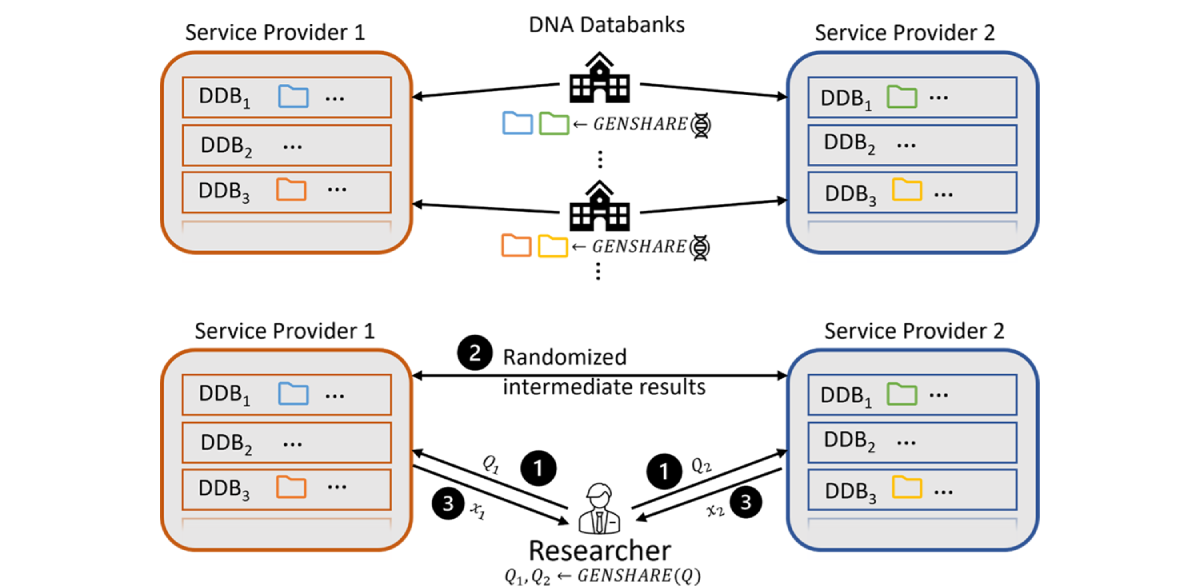
Network setup. The institutions first share their data (top) with the two service providers. The researcher can then query the database (bottom) and get the information they require for their research.

We had two different scenarios tested in our network setup, as shown in Figure 2. The first of which was a virtual network constructed for quick easy testing over a local area network. We then made use of CloudLab to host a real network. Using the virtual network, we ran our protocol on simulated data, and using the CloudLab network, we ran our methods on real-life data samples.

The virtual network was constructed using VirtualBox in the Ubuntu environment. We set up three virtual machines with access to the network and required the machines to talk on the network to communicate. The behavior of this network is consistent with that of a LAN. Using the three machines, we established a network and ran the protocols. Two machines provided input by secret sharing their data according to the protocol. The third machine helped to facilitate the Shamir and Mal-Shamir protocols. The third machine was not used during the Semi-OT and MASCOT executions.

For the CloudLab servers, we had three servers that could communicate with each other and provide computation. We used two of the servers as the input providers, and the third server helped to facilitate the realization of the Shamir and Mal-Shamir (three party) protocols. These servers better simulate a real-world computing environment.

### MP-SPDZ Setup

We used MP-SPDZ [9] for the implementation of the secure protocols. The MP-SPDZ documentation provides a comprehensive guide in setting up the experiments. In our setup, we had three servers *P*_0_, *P*_1_, *P*_2_. The servers *P*_0_, *P*_1_ were treated as users of the system, and they provided the input to the computation. This is because the two servers have their own privacy and security of interest, so there is less concern for collusion. If these two parties do not want to hide their information from each other, there is no reason to perform the computation. The last party *P*_2_ is there to obtain the necessary three parties for Shamir and Mal-Shamir. This party in practice would be an individual who is selected by both of the parties who provide an input to the computation as this can help ensure that the extra party is not biased toward one of the input parties.

## Results

We tested our protocols (ie, Shamir, Mal-Shamir, Semi-OT, and MASCOT) with simulated filtered genomic variants sets of up to 400,000 elements in length. Protocols were run over both a virtual network and a CloudLab network. The Shamir [22,26-28] and Mal-Shamir (maliciously secured by verification techniques by Chida et al [29]) protocols require third-party computation. The Semi-OT and MASCOT [19] protocols require at least two parties. In general, the honest majority is more efficient than the malicious majority protocols but at a minimum requires three parties to execute.

**Figure 3:**
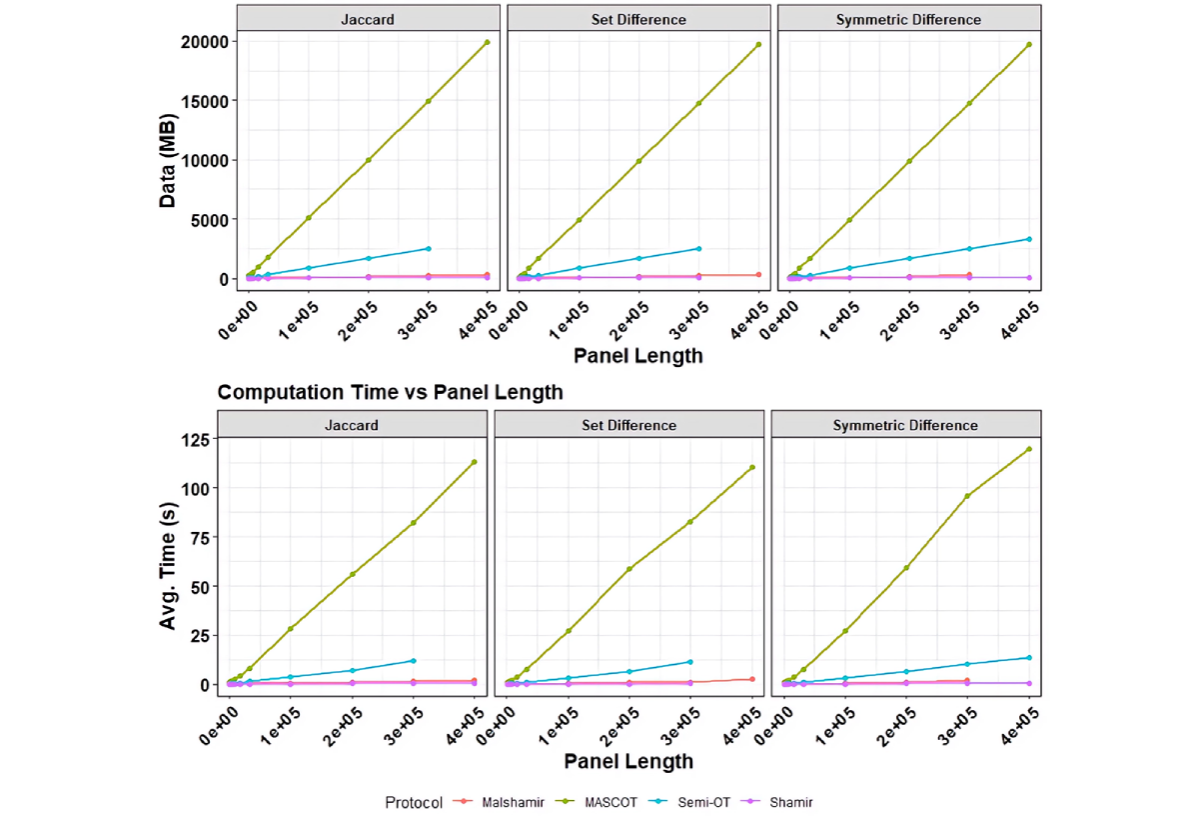
Each figure shows the growth of complexity as the array length grows. While we chart Mal-Shamir, MASCOT, Semi-OT, and Shamir together in the same chart, they are not comparable in terms of resource use alone. The more expensive protocols tend to have stronger security guarantees. However, the security guarantees of Shamir and Mal-Shamir are frequent enough for standard usage and are practical to use on larger sets. Mal-Shamir: Malicious Shamir; Semi-OT: Semihonest Oblivious Transfer.

We measure the resources needed to compute an operation (set difference, symmetric difference, and Jaccard similarity) between two sets over the specified length of the array through time and network communication. The unit of time measured here is in seconds as measured by the framework, and the network communication is measured in megabytes. Figure 3 shows these results over an array of panel sizes.

**Figure 4:**
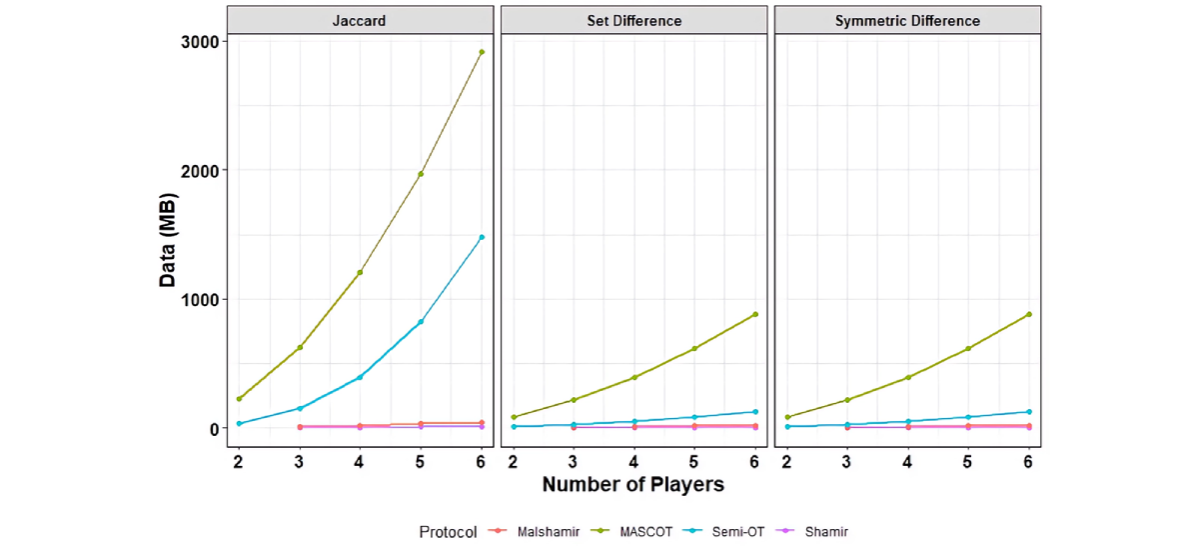
The communication complexity scales quadratically with the number of players. The benefit of increasing the number of computing servers is increased security.

We were able to calculate the Jaccard similarity over SNP panels of size 400,000 in 0.363 seconds with Shamir, 2.155 seconds with Mal-Shamir, 13.184 seconds with Semi-OT, and 113.397 seconds (about 2 minutes) with MASCOT. Shamir is the most efficient, whereas MASCOT takes the longest. The extra time MASCOT takes is due to extra security guarantees that it offers over the Shamir method. Figure 4 illustrates the growing complexity as the number of computing servers increases. Adding more servers has the benefit of improving the security of the system. When looking at Figure 4, one may notice that Shamir and Mal-Shamir do not have a statistic for two players. This is because these protocols do not support two players.

**Figure 5:**
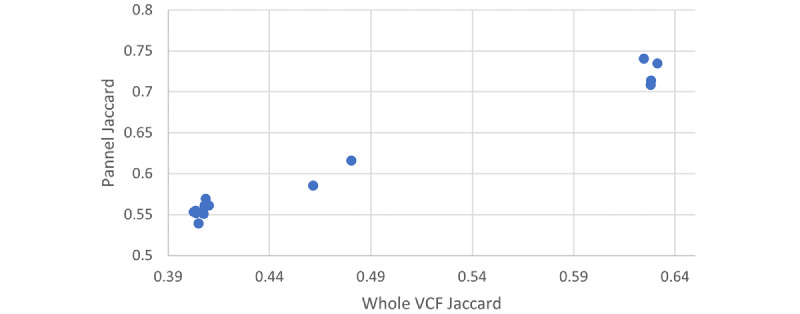
The Jaccard similarity is compared through filtered single nucleotide polymorphisms (Panel Jaccard) vs through the entire VCF using BEDTools (Whole VCF Jaccard). The correlation can be seen: HG002 matches HG003 and HG004 and HG005 matches HG006 and HG007. VCF: Variant Call Format.

We also ran our method on the GIAB data set [25]. The comparison required converting the VCF and SNP panel files into a binary array to be used in the secure computation. This process took on average 96.430 seconds (about 1 and a half minutes) per file using our custom code. This process is a one-time need, and the resulting files can be used multiple times for other comparisons. We used our secure method in conjunction with the SNP panel provided by Murray et al [10]. Figure 5 compares the Jaccard similarity between filtered SNPs and the entire VCF using BEDTools [11], which indicates a high correlation. For convenience, we reiterate that HG002 is the child of HG003 and HG004, and that HG005 is the child of HG006 and HG007. Then we ran different secure computing methods on the network. The costs of the computation with the panel size of 4763 are presented in Table 1.

The comparison by Jaccard similarity took an average of 31.008 seconds between two files. This comparison done by BEDTools assumes that the VCF files are sorted but does not require any preprocessing beforehand.

**Table 1:** This table shows the computation using the secure protocols from the filtered panel using 4763 single nucleotide polymorphisms after preprocessing.

Method	Time (s), average	Communication (MB)
Shamir	0.0350	1.671
Mal-Shamir^a^	0.1125	9.357
Semi-OT^b^	0.7085	40.570
MASCOT	5.4322	353.363

^a^Mal-Shamir: Malicious Shamir.

^b^Semi-OT: Semihonest Oblivious Transfer.

## Discussion

There are several established protocols in a secure multiparty computation that allow for computation over a finite field. We make use of a framework called MP-SPDZ that securely implements these operations as described in many secure settings.

Our work provides an efficient secure method for computing similarity between two genomic sequences by considering predefined variant panels. Our study only considers the presence of a variant (ie, binary representation) and does not explicitly compute the set based on the actual allele (ie, nucleotide identity A, T, C, G) or combination of alleles (ie, heterozygous positions) represented at that location. Although this is sufficient for most practical applications, our methods can easily be extended to compute the set based on the explicit allele presented at the given location.

Our method can easily be extended to allow only results beyond a certain threshold. Such a modification can be done by performing an inequality check at the end of any of the protocols. The inequality check only needs to be performed once and adds constant time to the protocol when the number of parties is fixed.

We presented four protocols that can be used to execute the arithmetical operations of our protocols. Based on the results in the previous section Shamir and Mal-Shamir are faster but have different security guarantees from Semi-OT and MASCOT. Mal-Shamir provides realistic security guarantees while requiring similar computational and network resources to Shamir. Thus, we recommend using Mal-Shamir to execute our protocols. Shamir requires at least three parties, so we suggest executing the protocol using Semi-OT when only two parties can be used. Though MASCOT provides malicious security, the method is still impractical on realistic data sets if we expect results in real time. However, in some situations it may be reasonable to allow processing over a day, then even MASCOT will be practical.

Our development may pave the way for a practical protocol to share human variant data securely, which may help support large-scale variant applications for precision medicine.

## Abbreviations

AWS: Amazon Web Services
GCP: Google Cloud Platform
GIAB: Genome in a Bottle
Mal-Shamir: Malicious Shamir
MP-SPDZ: Multi-Party Secure, Privacy-Preserving, and Decentralized Zeus
Semi-OT: Semihonest Oblivious Transfer
SNP: single nucleotide polymorphism
SP: service provider
UCSC: University of California, Santa Cruz
VCF: Variant Call Format

## Data Availability

Some data were simulated using the methods described in the Data subsection of the Methods; other data include the Genome in a Bottle data set [25].
